# Combined Effects of Arsenic and Cadmium With Selenium on Oxidative Stress Indicators in Goldfish: Implications for Veterinary Toxicology

**DOI:** 10.1002/vms3.71025

**Published:** 2026-06-12

**Authors:** Sarina Hosseinkhani, Mahdieh Raeeszadeh, Behnam Salimi

**Affiliations:** ^1^ Faculty of Veterinary Sciences Sa.C. Islamic Azad University Sanandaj Iran; ^2^ Department of Basic Sciences Sa.C. Islamic Azad University Sanandaj Iran; ^3^ Department of Aquatic Animal Health and Disease Sa.C. Islamic Azad University Sanandaj Iran

**Keywords:** arsenic, cadmium, *Carassius auratus*, oxidative stress biomarkers, selenium

## Abstract

**Background:**

The individual and combined effects of toxic heavy metals, including arsenic (As) and cadmium (Cd) and their interactions with selenium (Se), play a crucial role in modulating bioaccumulation patterns and oxidative stress responses in aquatic organisms.

**Objectives:**

This study aimed to evaluate the effects of As, Cd and Se, individually and in combination, on tissue metal accumulation and oxidative stress biomarkers in goldfish (*Carassius auratus*).

**Methods:**

A total of 210 fish were randomly assigned to seven experimental groups with three replicates each and exposed to single or combined concentrations of As, Cd and Se via water for 14 days. At the end of the exposure period, tissue concentrations of metals were determined using inductively coupled plasma mass spectrometry (ICP‐MS). Oxidative stress biomarkers, including malondialdehyde (MDA), catalase (CAT) and superoxide dismutase (SOD), were assessed using spectrophotometric methods and commercial assay kits.

**Results and Conclusions:**

Body weight increased in all treatment groups compared to initial values. Co‐exposure to Se significantly reduced As accumulation, with tissue concentrations decreasing from 0.16 ± 0.02 mg/kg in the As group to 0.10 ± 0.009 mg/kg in the combined exposure groups (approximately 62 % reduction; *p* < 0.05). In contrast, Cd accumulation showed no significant difference between the Cd (1.06 ± 0.09 mg/kg) and Cd+Se (1.01 ± 0.07 mg/kg) groups (*p* > 0.05). A significant positive correlation was observed between As concentration and MDA levels across all groups, including the control (*p* < 0.01). Conversely, Se exhibited a negative correlation with MDA and a positive correlation with SOD, indicating its protective role against oxidative damage. Overall, the results demonstrate that combined exposure to heavy metals can produce variable toxicological interactions, ranging from antagonistic to potentially synergistic effects, depending on the type of metal and exposure conditions. These interactions are closely associated with oxidative stress responses, highlighting potential ecological risks to aquatic organisms and public health concerns associated with the consumption of contaminated fish.

## Graphical abstract

Co‐exposure to arsenic and cadmium alters metal accumulation and oxidative stress in goldfish. Selenium reduces arsenic bioaccumulation and lipid peroxidation while enhancing antioxidant defences. Findings highlight metal‐specific interactions, with selenium offering partial protection, emphasizing ecological risks and the importance of monitoring combined heavy metal contamination in aquatic environments.

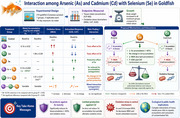

## 1 Introduction

The rapid development of industrial activities has led to the widespread release of heavy metals into aquatic and terrestrial ecosystems, resulting in significant environmental contamination, reduced food safety and both acute and chronic toxic effects in living organisms (Ali et al. 2019). Heavy metals are generally defined as elements with high atomic weight and density, which are naturally present in the Earth's crust but are increasingly mobilized through anthropogenic activities such as mining, industrial discharge, agriculture and wastewater effluents.

Due to their persistence, non‐biodegradability and strong affinity for biological macromolecules, heavy metals readily enter food chains and accumulate in plant and animal tissues, ultimately reaching humans through contaminated water, food and air (Jaishankar et al. 2014). Chronic exposure to these metals has been associated with a wide range of adverse health outcomes, including carcinogenicity, neurotoxicity, endocrine disruption and oxidative damage at the cellular level (Briffa et al. 2020).

Among these elements, arsenic (As), cadmium (Cd), mercury (Hg) and lead (Pb) are considered highly toxic even at low concentrations (Genchi et al. 2020). Cd exposure, primarily through food consumption, cigarette smoke and industrial emissions, leads to long biological half‐life and accumulation in vital organs such as the liver and kidneys, resulting in renal dysfunction, skeletal damage and increased cancer risk (Budi et al. 2024). As exposure, mainly via contaminated drinking water and agricultural products, is associated with gastrointestinal disturbances in acute cases and skin lesions, cardiovascular disorders and various cancers under chronic exposure conditions (Barchielli et al. 2022).

In contrast, selenium (Se) is an essential trace element required for antioxidant defence, immune function and thyroid hormone metabolism; however, its narrow margin between nutritional requirement and toxicity makes both deficiency and excess clinically relevant (Rayman 2012). At physiological levels, Se exerts protective effects through selenoproteins involved in redox regulation, whereas excessive intake may lead to selenosis, characterized by gastrointestinal, hepatic and neurological disorders.

A key toxicological mechanism shared by many heavy metals, particularly Cd and As, is the induction of oxidative stress through excessive production of reactive oxygen species (Raeeszadeh et al. 2024). This leads to lipid peroxidation, protein oxidation and DNA damage. Oxidative stress biomarkers such as malondialdehyde (MDA), along with antioxidant enzymes including superoxide dismutase (SOD), catalase (CAT) and glutathione peroxidase (GPx), are widely used to evaluate cellular redox imbalance in toxicological studies (Sies et al. 2022; Ghafarifarsani et al. 2024).

Fish are widely accepted as sensitive bioindicators of aquatic pollution due to their direct exposure to waterborne contaminants. In particular, goldfish (Carassius auratus) has been extensively used as a model organism in toxicology and environmental biology due to its physiological sensitivity, ease of maintenance and well‐characterized biochemical responses (Hong and Zha 2019).

Although previous studies have examined single‐metal toxicity, there remains a significant gap in understanding the interactive effects of combined exposure to As, Cd and Se. These interactions may be antagonistic or synergistic depending on concentration and exposure conditions. Se, in particular, has been reported to modulate the toxicity of heavy metals by altering their bioavailability, promoting the formation of metal–Se complexes and regulating oxidative stress pathways (Zwolak 2020; Uddin et al. 2024).

Therefore, the main objective of this study was to investigate the individual and combined effects of As, Cd and Se on bioaccumulation patterns and oxidative stress responses in C. auratus. Specifically, this study aimed to evaluate tissue metal accumulation and key oxidative stress biomarkers to clarify the mechanistic interactions between these elements. This approach provides a clearer link between exposure conditions and physiological outcomes, contributing to a better understanding of metal toxicity interactions in aquatic organisms and their potential implications for environmental and human health.

## 2 Materials and Methods

The selection of metal concentrations was based on previously reported 96‐h median lethal concentration (LC50‐96 h) values for As, Cd and Se in freshwater fish species, which are commonly used to establish sublethal exposure levels in aquatic toxicology studies. Sublethal exposure is typically defined as a fraction (commonly 1/10 or lower) of the LC50 value to avoid acute mortality while inducing measurable physiological and biochemical responses (Sprague 1973).

Based on a review of relevant toxicological literature, exposure concentrations equivalent to approximately 10% of reported LC50 values were selected to ensure biologically relevant but non‐lethal stress conditions. Accordingly, concentrations of 4 mg/L for As, 2.8 mg/L for Cd and 2 mg/L for Se were chosen to induce oxidative and biochemical responses without causing acute toxicity (Monteiro et al. 2009; Kavitha et al. 2010; Ghafari Farsani et al. 2015).

This approach is consistent with established experimental designs in aquatic toxicology, where sublethal metal exposures are used to evaluate oxidative stress biomarkers, enzyme activity alterations and bioaccumulation patterns (Grosell et al. 2006).

All biochemical substances and metal salts (Sodium arsenite, Sigma‐Aldrich, Lot No: 104266100; Cd chloride, Sigma‐Aldrich, Lot No: 210527; and sodium selenite, Sigma‐Aldrich, Lot No: 1203690) were obtained from certified analytical‐grade suppliers and prepared according to the manufacturers’ specifications to ensure consistency and experimental reliability.

### 2.1 Experimental Study Design

A total of 210 C. auratus with a mean body weight of 20 ± 8 g were obtained in spring 2024 from a commercial fish farm in Gilan Province, Iran and transported to the Laboratory of the Faculty of Veterinary Medicine, University of Kurdistan, Sanandaj. Upon arrival, fish were acclimated and randomly allocated to 21 glass aquaria (40 L each) under controlled laboratory conditions (20 ± 2°C; pH 7.5–8.5) with continuous aeration.

It should be noted that fish were evenly distributed among aquaria, with each treatment consisting of three independent replicates. Treatments were applied at the aquarium level; therefore, the aquarium was considered the experimental unit. At the end of the 14‐day exposure period, fish were randomly sampled from each aquarium, and measurements were averaged per tank to avoid pseudoreplication. These mean values were used as independent replicates in the statistical analysis (*n* = 3 per treatment).

Prior to the main experiment, the 96‐h median lethal concentrations (LC50) of As, Cd and Se were determined to define sublethal exposure levels. Fish were then exposed to 10% of the respective LC50 values for 14 days in separate treatment groups (three replicates per treatment). This sublethal exposure design is widely used in aquatic toxicology to assess physiological and biochemical responses without causing acute mortality (S. Eroglu et al. 2015; M. A. Aldoghachi 2016; J. Choi et al. 2022; M. Rabbane et al. 2022).

At the end of the exposure period, fish were collected, mortality was verified and biometric measurements were recorded. Muscle tissues were subsequently excised for biochemical and metal accumulation analyses.

### 2.2 Sample Collection and Preparation for Analysis

All solutions were prepared using analytical‐grade reagents. Throughout the study, ultrapure double‐distilled water was used. Nitric acid and hydrogen peroxide (H_2_O_2_) of supra‐pure quality were obtained from Merck (Darmstadt, Germany). Standard solutions of As, Cd and Se (1000 mg/L) were purchased from Sigma–Aldrich (St. Louis, USA) and diluted appropriately before use.

Muscle samples (∼10 g) were collected from the dorsal white muscle using a sterile surgical blade. Immediately after sampling, tissues were placed in sterile containers on dry ice to maintain the cold chain and then transported to the laboratory for further processing. All samples were analysed within one week of collection, well before their expiration date. Before analysis, samples were dried, crushed and ground, and 5 g of the resulting powder was used for measurements. Metal concentrations were expressed based on the dry weight of the samples.

Inductively Coupled Plasma Mass Spectrometry (ICP‐MS) was employed to quantify heavy metals. ICP‐MS is a sensitive analytical technique in which the sample is atomized and ionized by an inductively coupled plasma. The resulting atomic and small polyatomic ions are detected and quantified, allowing precise measurement of metals and certain non‐metals at trace levels. ICP‐MS can also differentiate isotopes of the same element, making it suitable for isotopic studies. Compared with atomic absorption spectroscopy, ICP‐MS offers higher sensitivity, precision and throughput.

For sample digestion, Pyrex glass tubes (Foss, USA) were used. Each tube was pre‐cleaned with 10% nitric acid for 48 h, followed by thorough rinsing with double‐distilled water to minimize external metal contamination. Approximately 0.2 g of each sample was digested with 3 mL of 70% nitric acid (Merck) and 1 mL of 30% H_2_O_2_ at 160°C for 8 h, until complete tissue dissolution. The digestion temperature was gradually increased from 50°C to 160°C. After cooling, the digested solutions were filtered through Whatman No. 1 filter paper, transferred into labelled containers, and diluted to 10 mL with double‐distilled water. The prepared samples were then analysed using a SCIEX ELAN DRC II ICP‐MS (PerkinElmer).

Metals were calibrated using a multi‐element standard (IV‐ICPMS‐71A, Inorganic Ventures) diluted to 0.05 µg/mL in 19.6% (w/w) nitric acid, matching the acid content of the samples. Six‐point calibration curves were constructed for each analyte, covering concentrations from the respective LOD to 200 ng/g. Measurements were conducted in full quantitative mode, with all calibration curves showing excellent linearity (*R* ^2^ ≥ 0.9999). Polyatomic interferences were evaluated by measuring multiple isotopes and verifying isotopic ratios in the digested samples (Rito et al. 2022; Poopak et al. 2024).

### 2.3 Preparation of Tissue Homogenates and Stress Oxidative Biomarkers

Approximately 0.5–1 g of dorsal white muscle tissue was excised, washed with cold phosphate‐buffered saline (PBS, pH 7.4), and immediately placed on ice. The tissue was then homogenized in 4–5 mL of cold PBS using a glass–Teflon homogenizer. The homogenate was centrifuged at 10,000 × *g* for 15 min at 4°C, and the supernatant was collected for biochemical analyses (Dias et al. 2020).

#### 2.3.1 Catalase Activity

CAT activity was determined spectrophotometrically by measuring the rate of H_2_O_2_ decomposition at 240 nm. The decrease in absorbance over time was used to calculate enzyme activity following standard protocols (Hadwan and Kadhum Ali 2018).

#### 2.3.2 Superoxide Dismutase Activity

SOD activity was measured based on its ability to inhibit the photochemical reduction of nitroblue tetrazolium (NBT). The reaction was initiated under light exposure, and the reduction of NBT was monitored at 560 nm, where lower absorbance indicates higher SOD activity (Weydert and Cullen 2010).

#### 2.3.3 Malondialdehyde Level

Lipid peroxidation was assessed by measuring MDA levels using the thiobarbituric acid reactive substances (TBARS) assay. Samples were incubated at 95°C for 30 min, and the resulting chromogen was measured spectrophotometrically at 532 nm (De Leon and Borges 2020).

#### 2.3.4 Protein Normalization

All enzymatic activities and MDA levels were normalized to total protein concentration in tissue homogenates, which was determined using the Bradford colourimetric method (Raeeszadeh et al. 2023).

### 2.4 Data Analysis

All statistical analyses were conducted with the aquarium considered as the experimental unit, and tank‐level clustering was incorporated into the study design. Measurements obtained from fish within each tank were averaged, and these mean values were used for all analyses (*n* = 3 per treatment) to avoid pseudoreplication.

Prior to analysis, data distribution and assumptions were evaluated. Normality was assessed using the Shapiro–Wilk and Kolmogorov–Smirnov tests, while homogeneity of variances was examined using Levene's test. When the assumptions for parametric analysis were satisfied, one‐way analysis of variance (ANOVA) was applied to compare quantitative variables among groups, followed by Tukey's post hoc test for multiple comparisons. Paired *t*‐tests were used, where appropriate, to assess within‐group differences (e.g., pre‐ and post‐intervention weights). Pearson correlation coefficients were calculated based on tank‐level mean values to evaluate associations between variables. Data visualization was performed using box plots, scatter plots and line graphs. Statistical analyses were conducted using SPSS (v22), GraphPad Prism (v10) and R (v4.4.1), with the level of significance set at *p* < 0.05.

## 3 Results

In Table 1, the survival rate of the different groups is presented. The highest survival rate was observed in the control group, while the lowest was recorded in the As group. However, the differences between groups were not statistically significant.

**TABLE 1:** Survival rate of the different groups during the experimental study.

	Control	As	Cd	Se	As+Se	Cd+Se	As+Cd+Se
Survival %	90.00 ± 7.89	80.31 ± 5.79	84.27 ± 7.80	87.36 ± 10.27	84.20 ± 6.80	81.50 ± 9.46	80.47 ± 8.93

**FIGURE 1:**
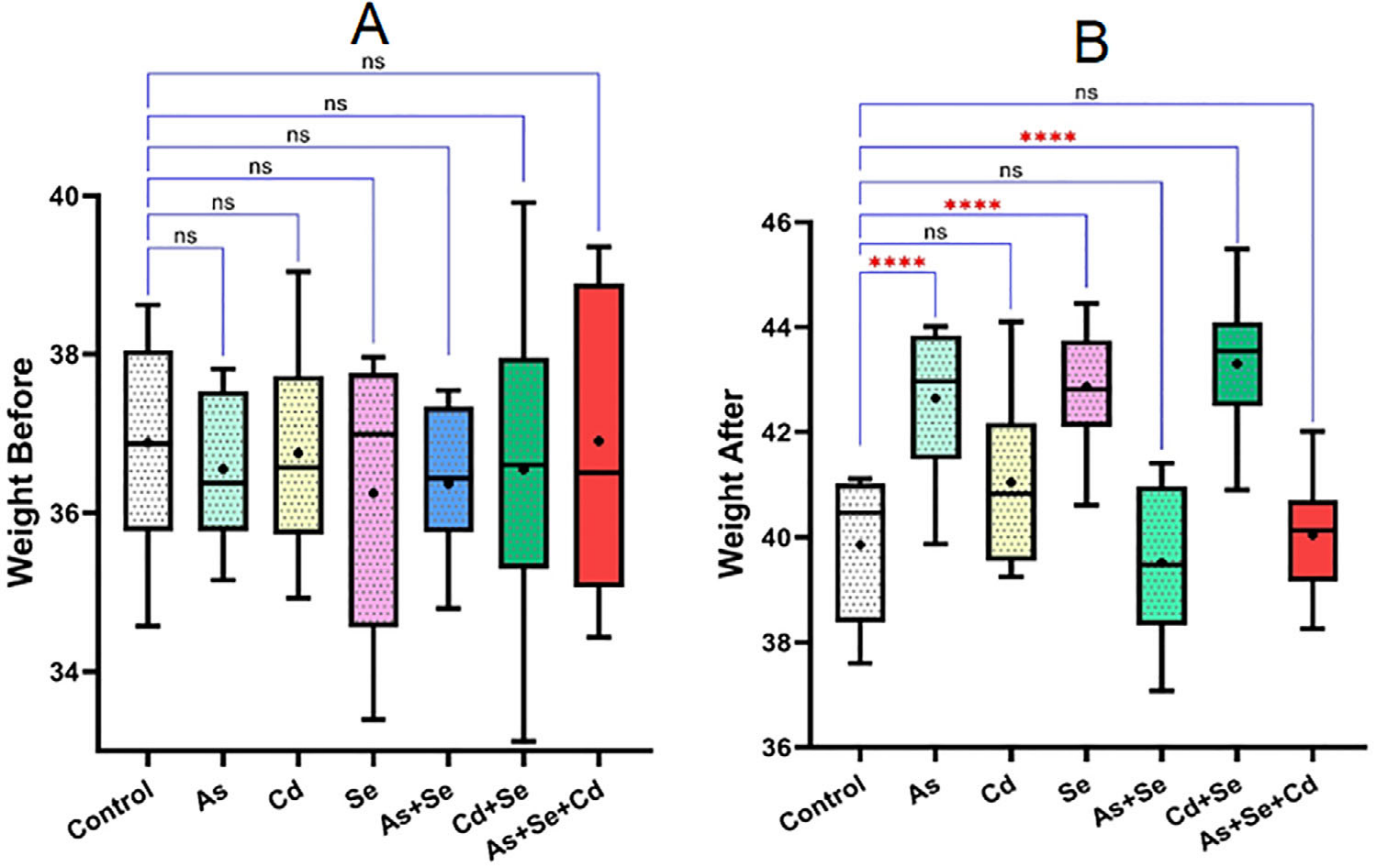
Comparison of initial and final weights of animals in the different study groups. ns, Not significant; ****p < 0.0001.

Figure 1 presents the initial and final body weights of the experimental groups. The results indicate no significant differences in initial mean body weights among the groups (*p* > 0.05), confirming uniformity at the start of the experiment. In contrast, final body weights increased in all treatment groups compared to their respective initial values. Moreover, significant differences were observed among the groups in terms of final body weight (*p* < 0.001). The lowest mean final weight was recorded in the As+Se group, whereas the highest final body weight was observed in the Cd+Se group.

**FIGURE 2:**
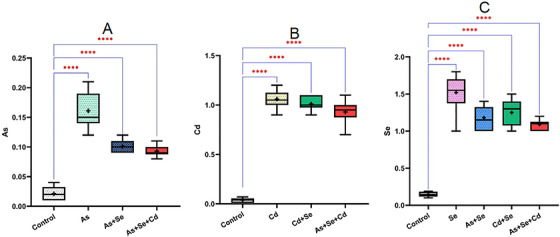
Concentration of heavy metals arsenic, cadmium and selenium in the different experimental groups, ****p < 0.0001.

The results regarding the concentrations of the toxic heavy metals As, Cd and Se in the experimental groups are presented in Figure 2. Accordingly, the data indicate that muscle As accumulation was lowest in the control group and was significantly altered following exposure to Se and Cd (*p* < 0.001) (Figure 2A). Cd concentrations were also lowest in the control group, while a reduction in Cd accumulation was observed in the As+Se treatment group (Figure 2B). In addition, Se levels showed a significant decrease following exposure to both Cd and As compared with the control group (Figure 2C).

**FIGURE 3:**
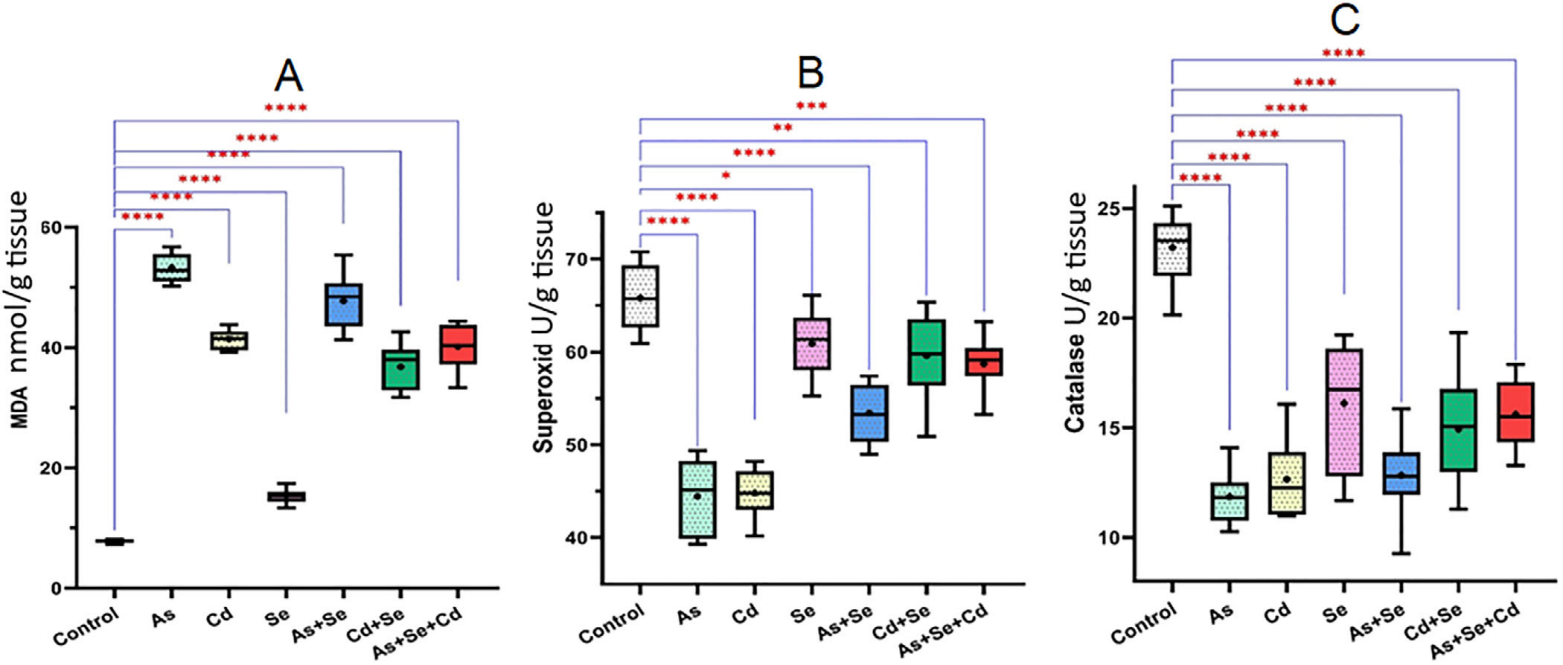
Comparison of oxidative stress parameters across the studied groups. *p < 0.05, **p < 0.01, ***p < 0.001, ****p < 0.0001.

The levels of oxidative stress biomarkers in muscle tissue across the experimental groups are presented in Figure 3. The results indicate significant variations among the groups.

As shown in Figure 3A, the mean MDA levels (nmol/g tissue) differed significantly among the experimental groups. The lowest MDA levels were observed in the control and Se groups. In contrast, a statistically significant increase in MDA concentration was observed in the As+Cd+Se group compared with the control group (*p* < 0.0001).

Figure 3B presents the mean activity of SOD (U/g tissue) across the different groups, revealing a significant difference among treatments (*p* < 0.001). The highest SOD activity was recorded in the control and Se groups, whereas the lowest activity was observed in the As and Cd groups. However, Se supplementation in the As and Cd groups resulted in an increase in SOD antioxidant activity.

Similarly, the mean CAT activity (U/g tissue) showed significant differences among groups (*p* < 0.001). The highest CAT activity was observed in the control and Se groups, respectively, while the lowest values were recorded in the As and Cd groups. Notably, CAT activity increased in the combined As+Se and Cd+Se groups, and this difference was statistically significant (*p* < 0.01) (Figure 3).

**FIGURE 4:**
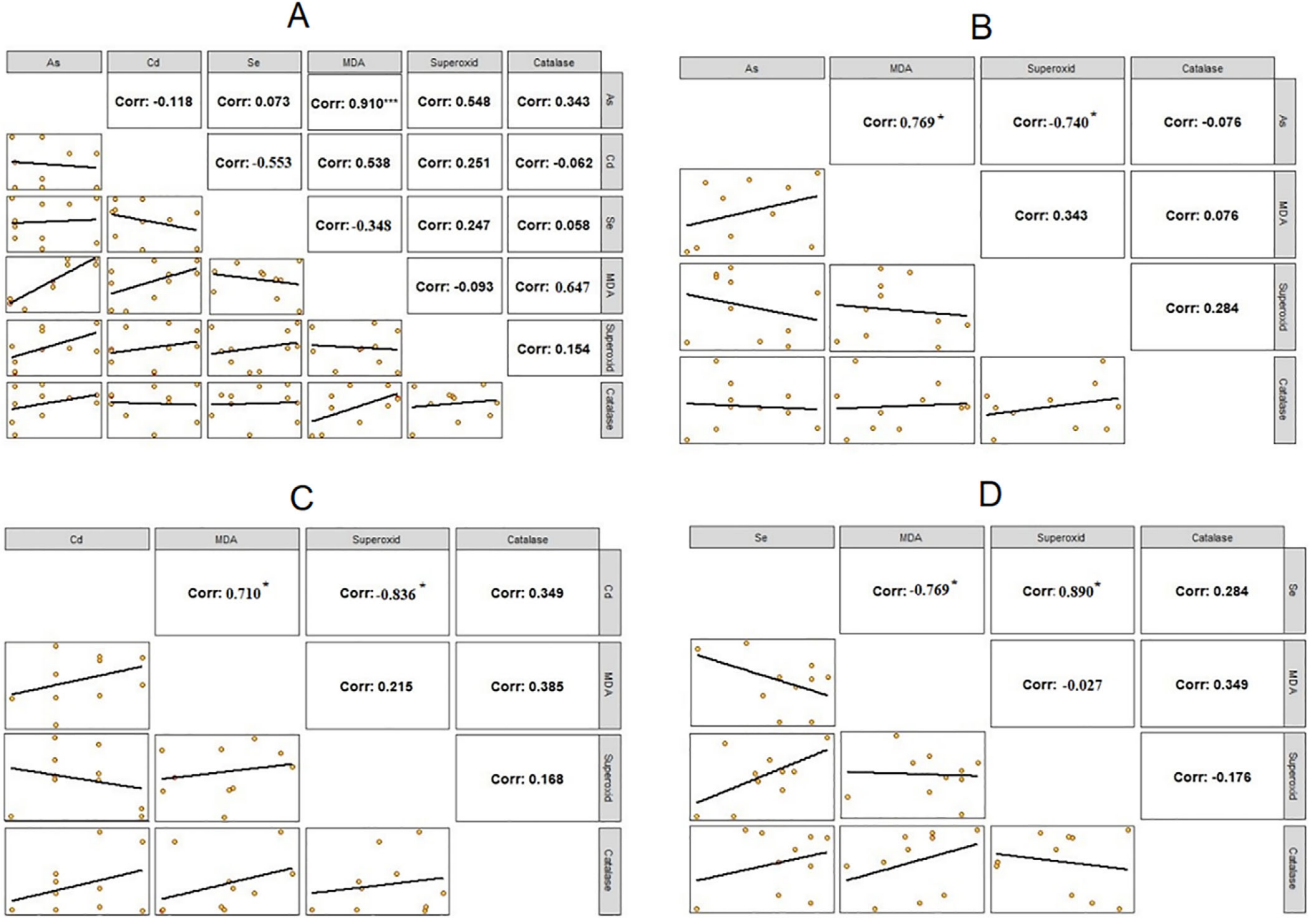
Correlation between heavy metal concentrations and oxidative stress markers in the control, As, Cd and Se groups.

The correlations between heavy metal concentrations (As, Cd and Se) in the independent treatment groups and oxidative stress biomarkers are presented in Figure 4. The results indicate distinct patterns of association between metal accumulation and biochemical responses.

Figure 4A shows that in the control group, a strong positive and statistically significant correlation was observed between As concentration and MDA (nmol/g tissue) (*r* = 0.910, *p* < 0.001). In contrast, the correlation between Se concentration and MDA (nmol/g tissue) was negative but not statistically significant (*r* = −0.348, *p* > 0.05). Similarly, correlations between Se and Cd concentrations were negative and not statistically significant (*p* > 0.05).

Figure 4B illustrates that in the As‐exposed group, As concentration showed a positive correlation with MDA (nmol/g tissue) (*r* = 0.769, *p* < 0.05), while exhibiting a negative correlation with SOD activity (U/g tissue) (*r* = −0.740, *p* < 0.05). The correlation between As concentration and CAT activity (U/g tissue) was negative (*r* = −0.076), but not statistically significant (*p* > 0.05).

Figure 4C demonstrates that in the Cd group, Cd concentration was positively correlated with MDA (nmol/g tissue) (*r* = 0.710, *p* < 0.05) and negatively correlated with SOD activity (U/g tissue) (*r* = −0.836, *p* < 0.05).

Finally, Figure 4D shows that Se concentration exhibited a significant positive correlation with SOD activity (U/g tissue) (*r* = 0.890, *p* < 0.05) and a significant negative correlation with MDA (nmol/g tissue) (*r* = −0.769, *p* < 0.05).

**FIGURE 5:**
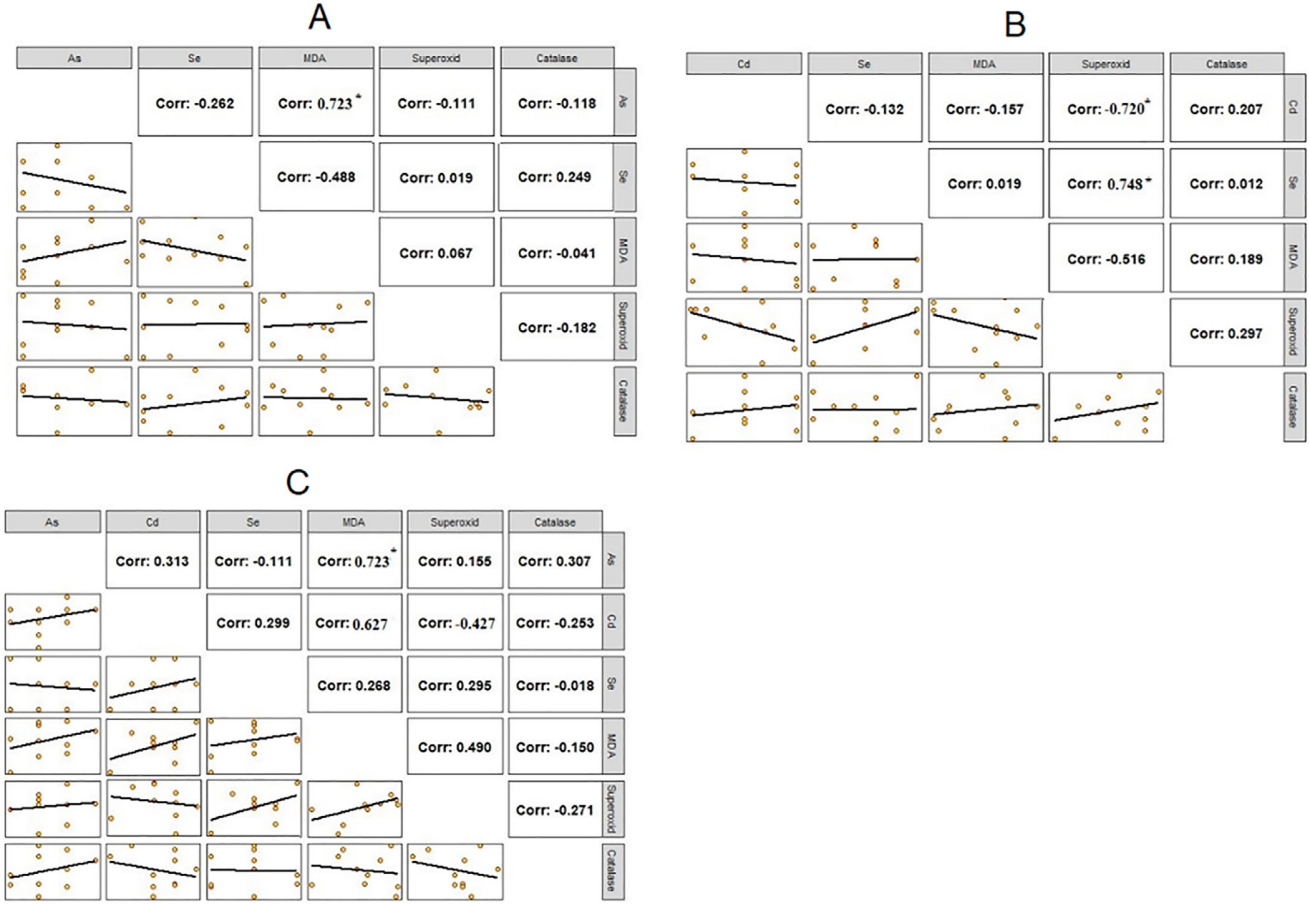
Correlation between heavy metal concentrations and oxidative stress markers in the As+Se, Cd+Se and As+Cd+Se groups.

Figure 5 presents the correlations between oxidative stress biomarkers and heavy metal concentrations (As and Cd) in the presence of Se. The results indicate distinct interaction patterns among treatments.

Figure 5A shows that in the As+Se group, As concentration exhibited a negative correlation with CAT activity (U/g tissue) and SOD activity (U/g tissue), while showing a positive correlation with MDA (nmol/g tissue) (*r* = 0.723, *p* < 0.05), which was statistically significant. In addition, Se concentration showed a positive correlation with CAT activity (U/g tissue) (*r* = 0.249) and SOD activity (U/g tissue) (*r* = 0.019), and a negative correlation with MDA (nmol/g tissue) (*r* = −0.488); however, these correlations were not statistically significant (*p* > 0.05).

Figure 5B demonstrates that in the Cd+Se group, Se concentration showed a significant positive correlation with CAT activity (U/g tissue) (*r* = 0.748, *p* < 0.05), while Cd concentration exhibited a significant negative correlation with SOD activity (U/g tissue) (*r* = −0.720, *p* < 0.05).

Figure 5C indicates that in the As+Cd+Se group, Cd concentration was negatively correlated with CAT activity (U/g tissue) (*r* = −0.253) and SOD activity (U/g tissue) (*r* = −0.427), while showing a positive correlation with MDA (nmol/g tissue) (*r* = 0.627). In addition, As concentration showed a positive and significant correlation with MDA (nmol/g tissue) (*r* = 0.723, *p* < 0.05).

## 4 Discussion

One of the major concerns associated with the consumption of aquatic organisms is the accumulation of heavy metals in their muscle tissues (Garai et al. 2021). This accumulation may not only adversely affect aquatic organisms—potentially leading to behavioural alterations, impaired growth and increased mortality—but also pose risks to human health due to the persistence and bioaccumulative nature of these metals (Garai et al. 2021). It has been suggested that metal accumulation in fish tissues is influenced by multiple factors, including metal concentrations in water as well as environmental parameters such as temperature, pH, salinity and hardness (Menon et al. 2023). In this context, the present study aimed to investigate the potential interactive effects of toxic and essential metals on oxidative stress responses under controlled experimental conditions.

The findings of this study may indicate cumulative and relatively non‐degradable effects of metals such as As and Cd. The observed modulation following Se exposure could suggest a mitigating role, as reflected by the possible reduction in MDA levels and enhancement of antioxidant enzyme activities. The significant correlation between As concentration and MDA levels in the control group might reflect underlying environmental exposure, possibly linked to background contamination sources such as well water. This possibility is supported by previous reports indicating As contamination in water resources of western Iran (Raeeszadeh et al. 2023; Poopak et al. 2024).

Cd and As are recognized as important environmental toxicants, and organisms are often exposed to them in combination. However, their interactive toxicological effects remain incompletely understood. Metallothioneins (MTs), which are cysteine‐rich metal‐binding proteins, are known to play a key role in Cd detoxification, although their involvement in As toxicity may be more complex and less clear.

Previous reviews have emphasized that heavy metals such as Hg, Cd, Pb and As can bioaccumulate through aquatic environments and exert toxicity primarily via oxidative stress mechanisms, potentially leading to tissue damage in vital organs (Zahran et al. 2025). Similarly, it has been suggested that stress‐related proteins, including heat shock proteins, antioxidant enzymes and MTs, may be involved in cellular defence responses and could serve as biomarkers of metal exposure (Gora et al. 2025).

Some studies have reported increased MT levels following As exposure, whereas others suggest that pre‐exposure to Cd may not significantly alter As‐induced toxicity, indicating that metal–metal interactions may depend on exposure conditions and doses (Amalraj et al. 2017). In line with this, it has been proposed that the toxicity of metal mixtures cannot be reliably predicted based on single‐metal exposure data alone, with interactions ranging from additive to synergistic effects (Yoo et al. 2021). The reduced tissue accumulation observed in the present study under combined exposure (particularly As+Se) may therefore reflect possible interaction effects influencing uptake, distribution or detoxification processes (Magos et al. 1980; Wu et al. 2016).

The distribution of heavy metals in fish tissues may also be influenced by metabolic activity, with lower concentrations typically observed in muscle compared to organs such as the liver (Shahjahan et al. 2022). This pattern could be related to the role of proteins such as MTs in metal binding and detoxification (Aalami et al. 2022). In addition, changes in metal distribution during growth and adaptation may further contribute to these differences (Wu et al. 2016).

The observed reduction in Se concentrations in combined treatments compared to Se alone may suggest possible interactions between Se and other metals affecting its bioavailability or incorporation into tissues. Previous studies have indicated that Se may modulate the toxicity of Cd and As through mechanisms related to redox balance and antioxidant defence (Naz et al. 2023). It has also been proposed that different chemical forms of Se may exert distinct effects on antioxidant pathways (Jamwal 2018; Raeeszadeh et al. 2022). Furthermore, Se may interact with certain metals to reduce their toxicity, possibly through the enhancement of selenoprotein activities such as GPx (Liang et al. 2023).

The increase in MDA levels observed in treated groups may reflect enhanced lipid peroxidation and oxidative stress, whereas the decrease in SOD and CAT activities could indicate impairment of antioxidant defence systems. The comparatively improved status in Se‐treated groups may suggest a protective or modulatory role of Se against oxidative damage. Combined exposure to Se with Cd and As appeared to partially counteract oxidative stress, which may be associated with improved antioxidant enzyme activity and reduced lipid peroxidation.

Antioxidant enzymes such as SOD and CAT are critical components of cellular defence mechanisms against reactive oxygen species. SOD catalyses the conversion of superoxide radicals into molecular oxygen, thereby limiting oxidative damage (Hermes‐Lima 2004). MDA, as a marker of lipid peroxidation, is widely used to assess oxidative stress status. Changes in these biomarkers may therefore reflect the balance between pro‐oxidant and antioxidant processes (Martins et al. 2022).

Overall, the largely non‐significant variations observed in some metal concentrations may suggest the presence of possible antagonistic or interactive effects among metals, which could influence their bioaccumulation and toxicity patterns in fish tissues.

## 5 Conclusion

This study demonstrates that simultaneous exposure to heavy metals, including As, Cd and Se, significantly influences tissue bioaccumulation patterns and biochemical responses in aquatic organisms. Se supplementation markedly reduced the tissue accumulation of both As and Cd, suggesting a modulatory and potentially antagonistic interaction. A consistent positive correlation was observed between As and Cd concentrations and MDA levels, whereas a negative correlation was found with antioxidant enzymes, particularly in the case of As, indicating pronounced oxidative stress induction.

The observed reduction in metal accumulation and oxidative damage in Se‐treated groups suggests a competitive and protective mechanism by which Se mitigates the toxic effects of As and Cd. However, the magnitude of these interactions appears to be influenced by several environmental and biological factors, including exposure dose, water quality parameters (e.g., pH and salinity) and species‐specific physiological characteristics, all of which can modulate metal uptake and toxicity.

Despite the valuable findings of this study, several limitations should be acknowledged. The present work is limited to biochemical endpoints in muscle tissue and does not include comprehensive molecular assessments. Future studies are recommended to incorporate gene expression analyses (e.g., RT‐PCR) of key antioxidant and stress‐related enzymes, as well as to evaluate additional target organs such as liver, kidneys and gills to provide a more complete toxicological profile. Moreover, extending the exposure duration is necessary to assess chronic toxicity and long‐term accumulation effects of heavy metals.

It is also suggested that future research include more comprehensive experimental designs with expanded treatment groups to better evaluate the interactive effects of As, Cd and Se under varying environmental conditions. Such integrated approaches would enhance the understanding of both individual and combined metal toxicity.

Overall, the findings of this study contribute to the ongoing scientific discussion on the interactive and cumulative effects of heavy metals and provide useful insights for environmental toxicology and aquatic risk assessment.

## Author Contributions

**Sarina Hosseinkhani**: conceptualization, data curation, formal analysis, investigation, methodology. **Mahdieh Raeeszadeh**: conceptualization, data curation, formal analysis, investigation, software, supervision, validation, visualization, methodology, writing – original draft. **Behnam Salimi**: conceptualization, data curation, formal analysis, investigation, methodology, writing – review and editing.

## Funding

The authors have nothing to report.

## Ethics Statement

All procedures followed international animal care guidelines and were approved by the Ethics Committee of Islamic Azad University, Sanandaj, with the code IR.IAU.SDJ.REC.1403.001. The study commenced after a 10‐day acclimatization period during which water parameters were monitored daily.

## Conflicts of Interest

The authors declare no conflicts of interest.

## Data Availability

The datasets used and/or analysed during the current study are available from the corresponding author upon reasonable request.
